# NAD^+^ Is a Food Component That Promotes Exit from Dauer Diapause in *Caenorhabditis elegans*

**DOI:** 10.1371/journal.pone.0167208

**Published:** 2016-12-01

**Authors:** Mykola Mylenko, Sebastian Boland, Sider Penkov, Julio L. Sampaio, Benoit Lombardot, Daniela Vorkel, Jean-Marc Verbavatz, Teymuras V. Kurzchalia

**Affiliations:** Max Planck Institute of Molecular Cell Biology and Genetics, Dresden, Germany; Scripps Research Institute, UNITED STATES

## Abstract

The free-living soil nematode *Caenorhabditis elegans* adapts its development to the availability of food. When food is scarce and population density is high, worms enter a developmentally arrested non-feeding diapause stage specialized for long-term survival called the dauer larva. When food becomes available, they exit from the dauer stage, resume growth and reproduction. It has been postulated that compound(s) present in food, referred to as the “food signal”, promote exit from the dauer stage. In this study, we have identified NAD^+^ as a component of bacterial extract that promotes dauer exit. NAD^+^, when dissolved in alkaline medium, causes opening of the mouth and ingestion of food. We also show that to initiate exit from the dauer stage in response to NAD^+^ worms require production of serotonin. Thus, *C*. *elegans* can use redox cofactors produced by dietary organisms to sense food.

## Introduction

The search for nutritional resources and selection of suitable food from a variety of available sources facilitate the adaptation of an organism to its environment. After finding of food, organisms evaluate its nutritive quality and decide whether to continue the search or to make use of the already detected food source [1].

The nematode *C*. *elegans* is widely used in research related to food detection and the physiological responses to different diets [2, 3]. In nature, *C*. *elegans* grows on ephemeral bacterial blooms, appearing randomly and seasonally in decomposing biological substances [4]. Under favorable environmental conditions, *C*. *elegans* undergoes a fast reproductive life cycle comprised of four larval stages (L1 to L4) that results in the formation of egg-laying adults [5]. In contrast, when the environment becomes harsh, worms enter a developmentally arrested non-feeding state specialized for long-term survival, called dauer [4, 6]. Dauers have a specific morphology, metabolism and are resistant to various kinds of stress. The body of dauers are radially shrunk, with a special impermeable cuticle and constricted non-pumping pharynx. The mouth (buccal cavity) is closed by a thick layer of body wall cuticle, which prevents dauers from feeding [7–9]. If environmental conditions improve and food becomes available, dauers open their mouth, start feeding, and resume the reproductive life cycle by molting into L4 larvae [6].

The presence of food *ad libitum* prevents dauer formation and stimulates recovery from the dauer state. Additionally, high density of the worm population antagonizes the effect of food by stimulating dauer arrest and inhibiting the recovery from dauer [6]. The increase of population density is sensed via dauer-inducing pheromones constitutively excreted by the worms [10, 11]. These pheromones, or “daumones”, have been characterized as a complex mixture of structurally varied hydrophilic ascarosides that exert their effects by binding to G-protein coupled receptors [12–15].

Despite extensive research on the chemical and biological effects of daumones, the identities of food components inhibiting dauer formation and inducing the exit from the dauer stage remain elusive. However, it had been found that extracts of microorganisms such as yeast and bacteria contain compounds (“food signal”), that inhibit dauer formation and, importantly, initiate dauer recovery [6, 11]. Herein, the food signal has been described as a labile, neutral and hydrophilic substance(s) with chromatographic and physical properties similar to low molecular weight nucleosides or carbohydrates. It has been also proposed that the food signal(s) could be a regular metabolite produced by many microorganisms [11].

In the current study, we show that NAD^+^ is a component of bacterial extract that promotes dauer exit under alkaline conditions. It causes opening of the mouth and ingestion of food. This process requires serotonin signaling. Thus, a redox cofactor produced by all living organisms can be used by *C*. *elegans* to detect the presence of food and to adapt its development accordingly.

## Results

### A quantitative bioassay for the purification of the food signal

**Fig 1:**
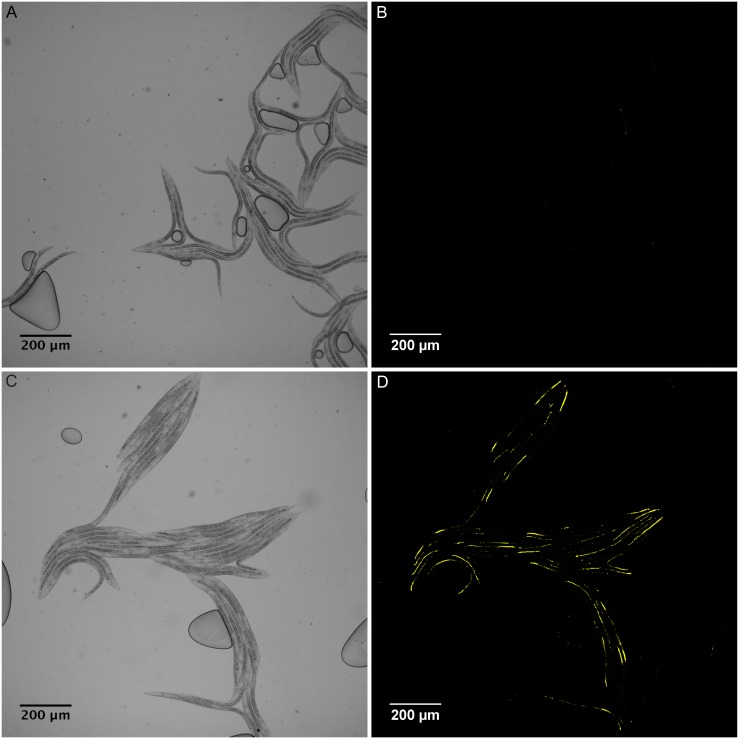
Bacterial extract (BE) initiates feeding in *C*. *elegans* dauer larva. (A) Bright field and (B) fluorescent (in shades of yellow) images of dauers incubated in water. (C) Bright field and (D) fluorescent images (in shades of yellow) of dauers incubated in the presence of BE.

The bacteria *Escherichia coli* is routinely used as food for *C*. *elegans* in laboratory conditions and it has been shown that dauer larvae exit diapause upon exposure to fresh *E*. *coli* in the growth medium [7]. In order to purify and chemically characterize the food signal derived from bacteria, we prepared a methanolic extract from *E*. *coli*. By phase partitioning we obtained two fractions—one enriched with hydrophilic metabolites and the other enriched with lipidic hydrophobic compounds (see Materials and Methods). Next, we developed a quantitative bioassay based on ingestion and accumulation of fluorescent microspheres in the gut of dauers that started to feed. Worms incubated with just solvent were absolutely devoid of microspheres in the gut, indicating that these worms failed to initiate dauer recovery (Fig 1A and 1B, for details see Materials and Methods). The hydrophobic phase of the extract did not show any gut staining and hence it was not investigated further. In contrast, when dauers were exposed to the hydrophilic phase of the bacterial extract, they displayed intensive fluorescent signal in the lumen of their guts (Fig 1C and 1D). From here on, we refer to this hydrophilic fraction as the bacterial extract (BE). The strong fluorescent signal of the microspheres allowed us to quantify the amount ingested (S1 Fig and Materials and Methods). The robustness of the assay and the effective recovery from the dauer stage promoted by the BE allowed us to proceed further with the isolation of the food signal.

### NAD^+^ promotes dauer exit

**Fig 2:**
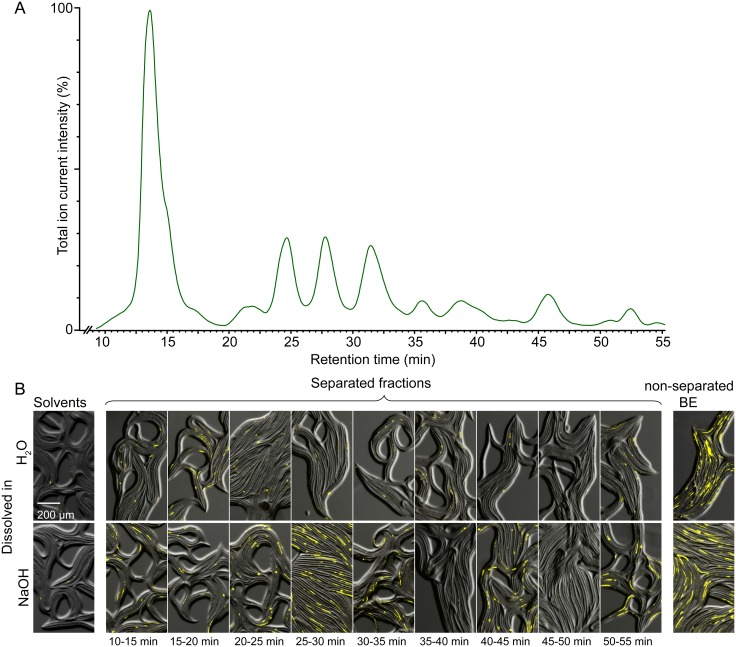
HPLC separation of the food signal activity. (A) HPLC-MS chromatogram of BE separated on a C18 column (ES-). (B) Representative composite images showing the bright field signal (in shades of gray) and fluorescence signal from ingested beads (in shades of yellow) of dauers incubated with HPLC fractions dissolved either in water or 5 mM NaOH solution.

We separated the active BE fraction using high performance liquid chromatography-mass spectrometry (HPLC-MS). A representative chromatogram is shown in Fig 2A. We collected five-minute fractions and tested them for their ability to induce recovery from the dauer stage (Fig 2B). Surprisingly, the activity of all fractions was very low (Fig 2B, upper row, H_2_O; S2 Fig, blue bars). Several reasons could explain the loss of activity: (a) the conditions of the assay after separation (e. g. pH, salt content, etc.) were different from the original, (b) synergistically acting components could be separated or (c) the active compound(s) could be lost during HPLC separation. We noticed that while the BE had a basic pH (pH~8.5–10), the separated fractions exhibited a neutral pH (pH~7). We hypothesized that the basic conditions might be required for the activity of the food signal compound(s). To restore the alkalinity, we dissolved the fractions in a 5 mM NaOH (pH around 11) solution instead of water. Remarkably, after this treatment one fraction displayed about 60% of the activity of the BE (Fig 2B, lower row, 5 mM NaOH, fraction 25–30 minutes; S2 Fig, red bars).

**Fig 3:**
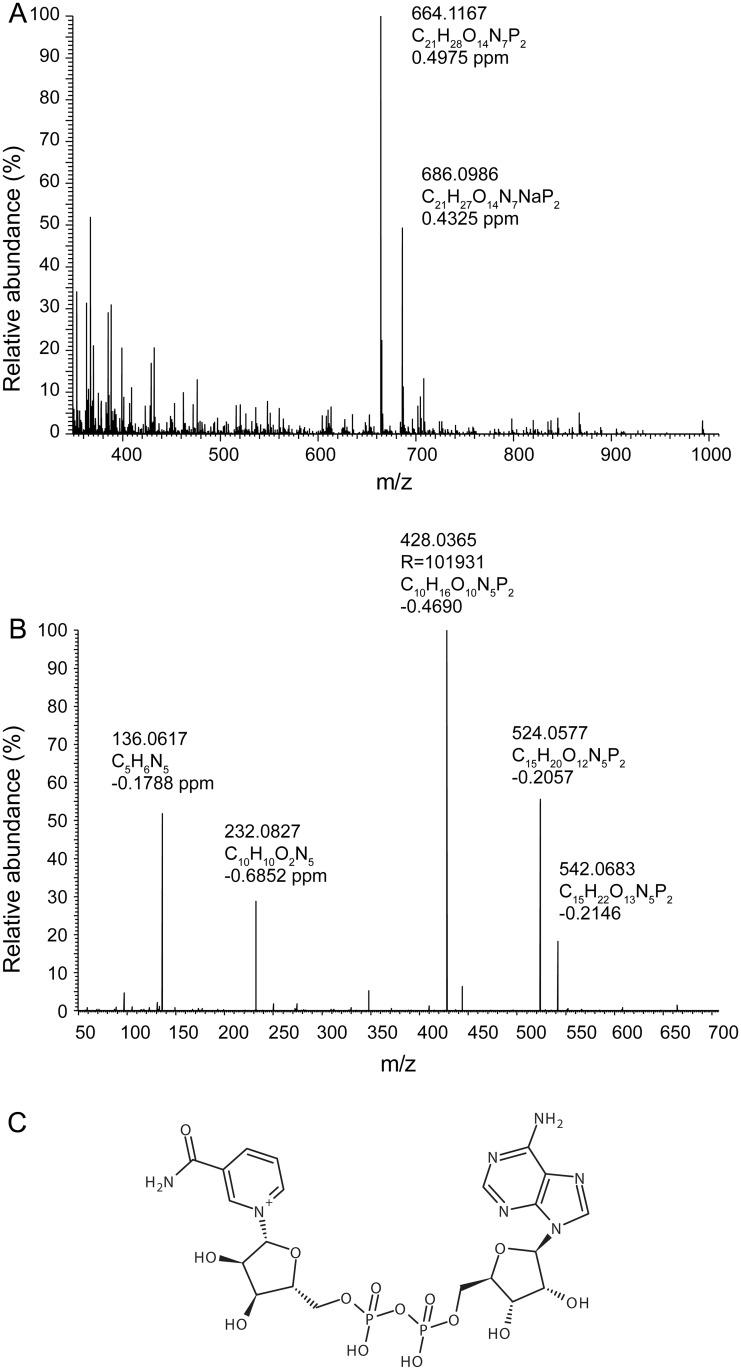
Active fraction of BE contains NAD^+^. (A) Mass spectrum of active HPLC fraction (minutes 25–30, ES+). (B) MS/MS of the most abundant ion and elementary composition of the fragments (ES+). (C) Structure of NAD^+^.

The chemical composition of this fraction was further analyzed by mass spectrometry, which revealed the presence of one prominent peak with an *m/z* of 664.1167 in the positive electrospray ionization mode (ESI+) (Fig 3A). Tandem mass spectrometry of the precursor ion revealed five main fragments with *m/z* of 542.0683, 524.0577, 428.0365, 232.0827 and 136.0617 (Fig 3B, ESI+). The resulting precursor mass and fragmentation spectra were matched with the METLIN metabolite fragmentation database (http://metlin.scripps.edu, MID: 101) [16]. The putative atomic composition and the fragmentation pattern matched perfectly with the coenzyme nicotinamide adenine dinucleotide (NAD^+^) (Fig 3C).

**Fig 4:**
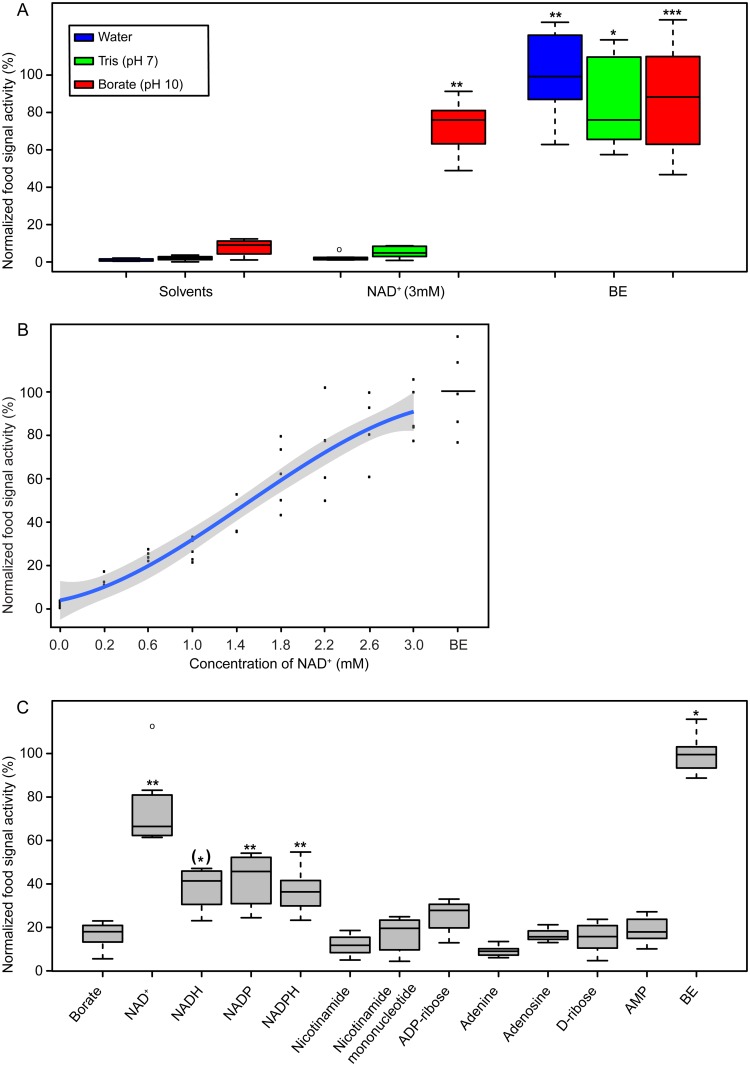
NAD^+^ has food signal activity at alkaline conditions. (A) Food signal activity of BE and 3 mM NAD^+^ in different solvents. All samples were compared with water as negative control. Average gut staining of wild type worms incubated in BE dissolved in water was considered as 100%. (B) Food signal activity of different NAD^+^ concentrations in borate buffer (pH 10). Individual data points (5 repeats per experiment) have been fitted using linear least squares regression to a 3-rd order orthogonal polynomial (blue line). Shaded areas around the smooth represent 95% confidence intervals. Average gut staining of wild type worms incubated in BE dissolved in borate buffer was considered as 100%. (C) Food signal activity of compounds similar to NAD^+^. All compounds were tested in 3 mM concentrations, dissolved in borate buffer (pH 10). All samples were compared with borate buffer (pH 10) as a negative control. Average gut staining of wild type worms incubated in BE dissolved in borate buffer was considered as 100%. Statistically significant differences are marked with asterisks: p ≤ 0.001, single asterisk; p ≤ 0.0001, double asterisk; p ≤ 0.00001, triple asterisk. An asterisk in round brackets indicates significance according to Bonferroni-Holm correction for multiple test comparison when the p-value does not lie within other significance intervals.

Next, we tested whether NAD^+^ indeed displays food signal activity. We performed the assay in neutral (Tris buffer, pH 7) and basic conditions (borate buffer, pH 10). The concentration of NAD^+^ was chosen from the literature, which stated the intracellular concentration of NAD^+^ in *E*. *coli* to be about 1 to 3 mM [17, 18]. As seen in Fig 4A, 3 mM NAD^+^ at alkaline conditions displayed comparable activity to BE. Similarly to the HPLC fraction of 25–30 minutes, NAD^+^ showed negligible activity at a neutral pH (Fig 4A). Moreover, we tested the activity at lower concentrations under alkaline conditions. Fig 4B shows a clear dose dependent activity of NAD^+^, with activity already detected in the high μM range.

The fact that NAD^+^ has activity only under alkaline conditions raises a possibility that its isomers produced upon speciation in aqueous solutions are active [19, 20]. The authors have separated some of these isomers using HPLC. We investigated whether commercially purchased β-NAD^+^, used throughout our study, undergoes changes that can be detected by LC-MS when dissolved in water. As seen in S3A Fig, we observed only a single peak with ions corresponding to NAD^+^. Activity was found only in the fraction containing this peak (S3B Fig). Thus, we think that the major activity is in β-NAD^+^, although we cannot exclude the presence of some minor isomers with identical masses.

Next, we asked whether the activity of NAD^+^ could be explained by its decomposition under alkaline conditions [21, 22]. We decided to test whether possible decomposition products are active. NAD^+^ was treated with 5 mM NaOH and the mixture was separated via HPLC. Only the peak corresponding to intact NAD^+^ displayed activity (S4 Fig), whereas its degradation products were inactive.

Taken together, we conclude that NAD^+^ is a component of bacterial extract that promotes dauer exit. However, our data suggest the existence of additional food signal compounds as, unlike NAD^+^, the BE displays activity at both a neutral and basic pH.

### NAD^+^, NADH, NADP^+^ and NADPH but not their building blocks induce the exit from the dauer stage

We next asked whether other coenzymes like NADH, NADP^+^ and NADPH also have the capability to induce dauer exit. Similarly to NAD^+^, these compounds were tested at a concentration of 3 mM in an alkaline environment. NADH, NADP^+^ and NADPH treated dauers also accumulated fluorescent microspheres in the gut lumen, although to a lower extent (~ two fold less, Fig 4C).

Theoretically, worms can metabolize NAD^+^ to various other compounds. Therefore, we tested the activity of the building blocks of NAD^+^: nicotinamide, nicotinamide mononucleotide, adenosine 5′-diphosphoribose (ADP-ribose), adenine, adenosine, D-(−)-ribose (D-ribose) and adenosine monophosphate (AMP). As shown in Fig 4C, none of these compounds displayed activity. Thus, from all compounds tested so far, only the intact redox dinucleotides displayed food signal activity.

### Exit from the dauer stage requires serotonin signaling

**Fig 5:**
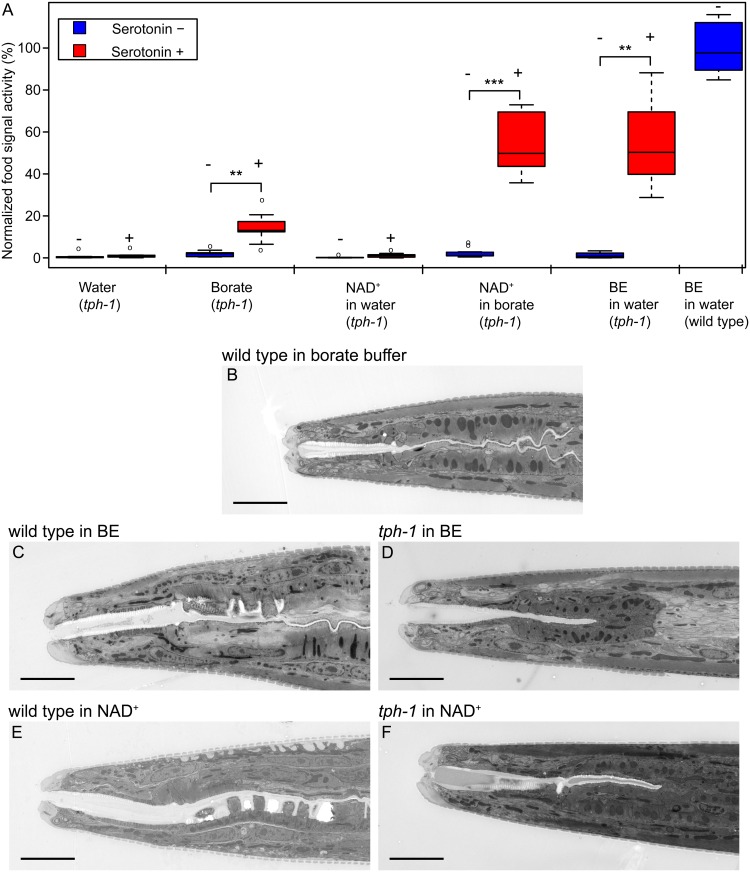
Food signal requires serotonin to initiate feeding. (A) Food signal activity of *tph-1(n4622)* mutant with or without 0.2 mM serotonin. NAD^+^ was at 3 mM concentration. (B) Representative images of the mouth in wild type dauers exposed to borate buffer, (C) BE in water and (E) NAD^+^ in borate buffer. (D) Representative images of the mouth in *tph-1(n4622)* dauers exposed to BE in water and (F) NAD^+^ in borate buffer. Scale bars show 5 μm in all micrographs. Statistically significant differences are marked with asterisks: p ≤ 0.0001, double asterisk; p ≤ 0.00001, triple asterisk.

So far, we have demonstrated that NAD^+^ promotes the dauers to start feeding. Next, we asked how does NAD^+^ activate this process. To feed, dauers need to unplug the buccal cavity and start pharyngeal pumping [6, 8]. The rate of pharyngeal pumping is determined by inputs from the nervous system and its regulation depends on the presence of food and production of the neuromodulator serotonin [23–25]. When food is abundant, serotonin is produced and this results in intensified pumping [26]. Therefore, the production of serotonin might be required for the induction of feeding in the dauer larvae. To test whether serotonin is required for the action of NAD^+^, we assayed the mutant *tph-1(n4622)*. This mutant bears a deletion in the gene encoding tryptophan hydroxylase, the enzyme that catalyzes the rate-limiting step in the serotonin biosynthesis [27]. *tph-1* dauers did not display fluorescent signals in their guts, independent of treatment with NAD^+^ or BE (Fig 5A). However, when supplemented with serotonin, *tph-1* dauers accumulated fluorescent microspheres in their guts in the presence of both NAD^+^ and BE (Fig 5A). Serotonin alone did not induce feeding (Fig 5A).

We asked whether the absence of fluorescence microspheres in *tph-1* might be due to the closed mouth (buccal cavity). Therefore, we performed electron microscopy of longitudinal sections of heads of wild-type and *tph-1* dauers. As expected, dauers remained with a sealed mouth upon treatment with solvent (Fig 5B). When treated with BE or NAD^+^, wild type dauers clearly showed an unsealed mouth (Fig 5C and 5E, S5 Fig). In the presence of BE, *tph-1* opened their mouths (Fig 5D and S5 Fig), but in contrast, *tph-1* dauers exposed to NAD^+^ exhibited a closed mouth (Fig 5F, S5 Fig). Serotonin alone also did not induce the opening of the mouth in *tph-1* dauers (S5 and S6 Figs). Taken together, these findings demonstrate that serotonin signaling promotes both buccal unsealing and pharyngeal pumping in response to NAD^+^. The opened mouth morphology in *tph-1* dauers exposed to BE suggests that additional components produced by bacteria can signal for mouth unsealing in a serotonin-independent manner. This reinforces the notion that the BE might contain molecules other than NAD^+^ harboring food signal activity.

## Discussion

As a major finding of this study, we show that NAD^+^ is able to promote exit from dauer diapause in *C*. *elegans*; dauers exposed to NAD^+^ open their mouths and initiate feeding. Additionally, NAD^+^ shows bioactivity only under alkaline conditions and requires serotonin signaling. NAD^+^ is one of the central metabolites of living organisms [28] and participates in many signaling processes [29–31]. It is astounding that nematodes can sense the presence of nutrition by detecting one of the major metabolites of all cells. Thus, NAD^+^ for bacterivore nematodes could be a universal signal for detecting their food (bacteria).

**Fig 6:**
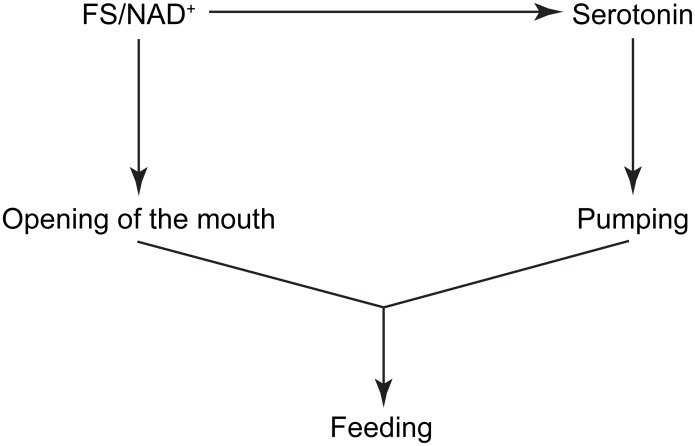
A scheme of dauer larva recovery caused by exposure to the food signal or NAD^+^. Food signal present in BE is sensed by the dauers, causes opening of the mouth and activation of serotonin signaling, the latter is necessary for pharyngeal pumping. Together, the opening of the mouth and pumping lead to food ingestion and subsequent dauer larva recovery.

Based on our observations, we suggest the following scheme for the activation of exit from the dauer stage by BE or NAD^+^ (Fig 6); first a food signal present in BE is sensed by dauers and causes opening of the mouth. Second, the food signal activates the serotonin pathway that is necessary for pharyngeal pumping. Together, the opening of the mouth and pumping leads to ingestion of food, which initiates the dauer larva exit (Fig 6).

A number of results suggest that other unidentified compounds also have food signal properties or at least assist the activity of NAD^+^. Firstly, as NAD^+^ displays food signal activity only at alkaline conditions, in contrast to BE, there might be other food signal components acting at a neutral pH. Interestingly, the food signal activity isolated by Golden and Riddle [11] behaved as a labile, neutral and hydrophilic compound. This unidentified substance(s) might act complimentarily to NAD^+^. Secondly, the *tph-1(n4622)* mutant strain opened the mouth only in the presence of BE but not NAD^+^. Finally, because other redox cofactors such as NADP^+^, NADPH, and NADH are also present in food, they might also contribute to the food signal.

In addition to mouth opening and the induction of pumping, the food signal should also remodel the major developmental pathways regulating the dauer stage: (steroid hormone, TGF-β and insulin) [32, 33]. Presently, we could only speculate how these processes can be activated.

The current study highlights the importance of pyridine nucleotides on dauer larva exit, but also raises some important questions: 1) Is NAD^+^ a metabolite that is capable of promoting the exit, but it is not a component of the natural food signal? and 2) Why is NAD^+^ only active under alkaline conditions? We think there are some indications that support NAD^+^ as a genuine food signal. Although the concentration range of activity might appear too high, as we mentioned previously, NAD^+^ can be part of multicomponent signal. Regarding the pH range of activity, it is known that *C*. *elegans* is attracted by a high pH [34], which can induce lysis of cells in bacterial blooms. Thus, in these regions NAD^+^ might be released in higher concentrations. A definite answer to these questions will be provided by delineating the molecular mechanisms of action, including the identification of the NAD^+^ receptor in *C*. *elegans* neurons, which we aim to clarify in further work.

## Materials and Methods

### Chemicals

Low-glucose Dulbecco’s modified Eagle’s medium (D-MEM), amphotericin B and streptomycin/penicillin were purchased from Life Technologies. HPLC grade methanol and hexane were purchased from Rotisolv and all other chemicals from Sigma-Aldrich, if not stated specifically.

### Worm and bacteria strains

*C*. *elegans* strains and *E*. *coli* NA22 were obtained from Caenorhabditis Genetics Center (CGC), which is funded by NIH Office of Research Infrastructure Programs (P40 OD010440).

### Culture conditions

*C*. *elegans* strains were propagated on NGM agar plates seeded with *E*. *coli* NA22 at 20°C [35].

Large amounts of dauers of all strains used in this study were generated by growing *C*. *elegans* eggs at 25°C in liquid culture [36] with antibiotics (50 mg/L streptomycin and 10 mg/L nystatin) [37] until they were arrested as dauers (10–14 days).

Dauer larvae were obtained by 1% SDS treatment of mixed stages of worms [7].

### Preparation of bacterial extract

*E*. *coli* NA22 strain was inoculated into 2 L of low-glucose D-MEM and incubated overnight at 37°C. The bacterial suspension was centrifuged at 4000 g for 20 min. The bacterial pellet was dissolved in 40 ml of methanol and incubated for 1 h in a glass tube (with shaking). The methanolic bacterial suspension was pelleted at 1100 g for 20 min and the supernatant was then taken and washed twice with 2 volumes of hexane in a separation funnel with glass plug (Lenz). The purified methanolic BE was dried with nitrogen flow and this dried BE was then dissolved in 2 ml of 50% methanol and filtered through syringeless 0.45 μm PTFE membrane HPLC filters (Whatman). The filtrate was placed into a 1 ml glass screw neck total recovery vial (Waters), dried with nitrogen flow and dissolved in 1 ml of water. The whole extraction procedure was performed in the dark and the BE was stored for a month at 4°C protected from light.

### *C*. *elegans* dauer larva recovery bioassay

Wild type dauer larvae, obtained from liquid culture, were incubated for 3 h in water or buffer to which a source of food signal was added. Around 1000 dauers were incubated in 1.5 ml micro tubes, in a total volume of 300 μl, kept at 25°C, 1050 rpm in a thermomixer (Eppendorf). The bioassay was performed in the presence of antibiotics (50 μg/ml kanamycin; 2.5 μg/ml amphotericin B; 100 μg/ml of streptomycin and 100 units/ml of penicillin). After 3 h of incubation, 0.8 μl of fluorescent microspheres (Fluoresbrite^™^ Polychromatic Red 0.5 Micron Microspheres) [38] were added to the worm suspension and, after 2.5 h, dauers were washed 3 times with 0.5% Tween 20 and twice with water. Dauers were then anesthetized with 20 mM sodium azide and subjected to microscopy analysis. In the bioassay, 50 μl of BE was added to 300 μl of the incubation mixture, which was used as a positive control. NAD^+^ was added externally up to a final concentration of 3 mM.

### Light microscopy

In all experiments, except the ones in which the activity of HPLC-separated fractions were assayed, worms were mounted on 2% agarose pads on top of glass slides (Thermo scientific, Superfrost Plus) and anesthetized with 20 mM sodium azide. All liquid was aspirated, after which some of the worms clustered together or remained single. Slides were then covered with 0.17 +/- 0.005 mm cover slips (Menzel-Glaeser) and the dauers were observed under a Multiphoton Laser Scanning Confocal Microscope (Zeiss LSM 780 NLO, Zeiss Plan-Apochromat 10x0.45 air objective, Laser DPSS 561 nm and halogen lamp for transmitted light, PMT (red) and T-PMT detectors).

Dauers used for detection of the food signal activity of HPLC-separated fractions (Fig 2, S2, S3 and S4 Figs) were placed on Petri dishes containing 1.5% agarose (with 20 mM sodium azide as an anesthetic) and visualized with Olympus SZX16 stereomicroscope (Olympus SDF PLAPO 1XPF objective, X-Cite 120PC Q series light source (120 W metal halide lamp), Ex530-550/Em575, Qimaging monochrome CCD camera, QCapture 2.90.1 software).

### Image analysis

Every image was captured in two channels: transmitted light (S1A and S1D Fig) and beads fluorescence (S1B and S1E Fig). In order to quantify the amount of microspheres ingested by the worms, an image analysis pipeline was designed. First, the area occupied by the worms (A_w_) was extracted (see S1C and S1F Fig**)**. The sum of the microsphere signals (see S1B and S1E Fig**)** was then calculated as the product of mean intensity (I_m_) and area (A_m_) of the fluorescent microspheres within the region occupied by the worms (see S1C and S1F Fig**)**. The ratio (I_m*_A_m_)/A_w_ expressed in arbitrary units was then used to compare the quantity of microspheres ingested by the worms (S1G Fig).

The bright field images were acquired with identical settings. For fluorescent images, the gain was globally adjusted in each experiment and kept constant within the same experiment, to avoid bead signal saturation. Each measure was then converted to a percentage (S1H Fig) by comparing it to the food signal activity of the experimental positive control (average food signal activity of BE in solvent), which was considered as 100%. If multiple solvents were used in the experiment, the solvent for which BE showed the maximum effect was considered for this normalization. For each sample, 4 to 13 images were considered, with up to 100 worms per image. For the detection of the activity of HPLC-separated fractions (Fig 2 and S2 Fig) 2 images, with up to 100 worms per image, were considered for each sample, as this was deemed enough to make a conclusion on the activity of each fraction.

The detection of the regions of the worms consisted of 4 steps. First a top hat filter was applied to remove the background signal (observed as a slowly varying signal), so that only the details smaller or equal in radius to the worm width were kept. This was achieved by setting the radius of the top hat filter to a value similar to the worm width. The remaining signal was blurred with a Gaussian filter so that the regions with worms appeared homogeneous in intensity; a Gaussian standard deviation of twice the worm width was chosen. A mask of worms regions was then obtained by thresholding the image with a mean intensity threshold. Additionally, at this stage, any small regions, i.e. smaller than a typical worm area, were filtered out. The area of the mask provided an estimate of the area occupied by the worm A_w_ (it was also proportional to the number of worms). Both worm width and the typical area of a worm could then be estimated from the images (using Fiji for instance) [39]. If necessary, unwanted regions were manually removed from the mask before measuring A_w_.

To determine the sum of fluorescence signal, a user set threshold was applied to the image of the microspheres. The thresholding value of the fluorescence channel was chosen once and used for all of the following experiments, such that the unwanted signal (background noise and worm autofluorescence) was not selected in the resulting mask. For all the results, except the analysis of HPLC separated fractions, we chose a threshold value of 1000. For the analysis of HPLC separated fractions (Fig 2 and S2 Fig) we chose a threshold value of 50, as these images were acquired with a different set-up and this lower threshold value was found to be optimal for these new settings. Next, the worm mask and microspheres mask were intersected so that we were able to define the microspheres inside the worms, therefore excluding any microspheres that were not washed and randomly distributed outside the worms’ guts. The sum of the microspheres image intensity was then measured providing an estimation of I_m*_A_m_.

This analysis was implemented as a plugin for the freely available Fiji software. The plugin was tested on current version of Fiji: (Fiji is just) ImageJ [39, 40] version 2.0.0-rc-49/1.51d. The code of the plugin is available under the following link: http://wiki.imagej.net/CElegansBeadsAnalysis_plugin.

### HPLC separation of BE and mass spectrometric detection of the food signal

HPLC was performed on an Agilent HPLC system (1200 Series Autosampler and Binary Pump, 1260 Series Infinity High Performance Degasser). Stable column temperature was maintained with a Jetstream 2 Plus column thermostat (40°C). The column flow was split between Waters LCT time-of-flight mass spectrometer with an electrospray ionization source for MS analysis and automatic Waters Fraction collector II (1:20). PEEK tubing with 0.25 mm inner diameter (Idex) was used during the separation. Mobile phase was filtered via 20 μm glass inlet filters (Agilent). LC-MS system was operated with MassLynx V4.1 software.

95 μl of BE was separated using Waters XTerra Prep MS C18 column (10 mm x 250 mm, 10 μm particle size) with XTerra MS C18 Prep Guard Cartridge (10 mm x 10 mm, 10 μm). A HPLC gradient from water to 30% methanol (both solvents containing 0.005% ammonium acetate) was used for 1 h with a flow rate of 1 ml per minute to separate injected BE. 5 min fractions were collected in glass tubes and evaporated in a vacuum concentrator. The dried pellets were then re-dissolved in 250 μl of water and kept at 4°C for further analysis.

MS detection was performed with a capillary voltage of 3000 V, cone voltage of 40 V, source temperature of 130°C and desolvation temperature of 120°C. The MCP detector was set to 2700 V. Nitrogen was used as a cone (100 L/h) and desolvation (350 L/h) gas. Mass spectra were acquired in both positive (+40 V) and negative (-20 V) ion modes in the 100–1000 atomic mass units *m/z* range. Before usage, the instrument was calibrated with orthophosphoric acid in positive and negative ion modes.

### Structural analysis of NAD^+^ containing fraction by tandem mass spectrometry

Characterization and structural elucidation of the NAD^+^ containing fraction was performed by MS analysis on a QExactive mass spectrometer (Thermo Fisher Scientific, Bremen). The fractions were dissolved in CHCl_3_/MeOH/iso-propanol (1:2:4, v:v:v) containing 7.5 mM ammonium acetate. The analyte was directly infused into the mass spectrometer at a flow rate of 200 nL/min using a robotic nanoflow ion source TriVersa (Advion BioSciences, Ithaca NY). Gas pressure and voltage were set to 1.25 psi and 0.95 mV, respectively. FTMS and tandem FTMS spectra were acquired in the positive and negative ion modes at R_*m/z* = 200_ = 140,000 in the Orbitrap analyzer. To acquire tandem mass spectra, precursor ions were isolated within *m/z* window of 1.0 Da and fragmented in higher collision energy (HCD) mode at the normalized collision energy of 20% in positive ion mode. MS/MS spectra were acquired on the Orbitrap analyzer with the targeted resolution of R_*m/z* = 200_ = 140,000.

NAD^+^ identification was confirmed by searching the obtained putative atomic composition as well as the tandem MS spectra online in METLIN (METabolite LINk) metabolite fragmentation database (http://metlin.scripps.edu, MID: 101) [16].

Chemical structures were created using ACD/ChemSketch, Freeware Version V10E41 [41].

### Electron microscopy

Worms were directly frozen without freezing additives with a high-pressure freezing unit (EMPACT2, Leica), followed by an automated freeze substitution (AFS2, Leica) in an acetone cocktail (containing 1% osmium tetroxide, 0.1% uranyl acetate and 0.5% glutaraldehyde) with a slope of +2.5°C/hour (from -90°C up to 0°C). Samples were rinsed with acetone, and then stepwise infiltrated with mixtures of acetone and Epon LX112-resin (Ladd Research) from 1/3 over 1/2 to 2/3 amount of resin (one hour each step) at room temperature. Samples were left in pure resin overnight and then for another four hours in fresh resin before mounting between slides and polymerizing at 60°C.

Longitudinal and planar oriented serial sections of 70 nm were taken with an Ultra microtome (Ultracut UCT, Leica), and post-contrasted in 1% uranyl acetate in 70% methanol, followed by lead citrate.

The sections were examined under an electron microscope (Philips Tecnai12, FEI) at 100 kV and photographs were taken with a TVIPS-camera (Tietz) [42, 43].

### Statistical analysis

Quantitative analysis of variations between data samples of different conditions was performed using a Mann-Whitney [44] test with a two-sided alternative hypothesis at 95% significance level. To avoid error accumulation during multiple comparisons, the Bonferroni-Holm method [45] was employed to assess significance, with individual tests performed as mentioned previously.

Statistical analysis was performed using the R software package version 3.1.3 [46].

## Supporting Information

S1 Fig BE initiates feeding in *C*. *elegans* dauer larva. Images the same as in Fig 1 were used to exemplify food signal activity quantification procedure. (A) Bright field and (B) fluorescent (in shades of yellow) images of dauers incubated in water. (C) Software analyzed image showing the area occupied by the dauers (red contour) and quantification of area and mean intensity of fluorescent beads inside the guts of dauers (green color contour) incubated in water. (D) Bright field and (E) fluorescent (in shades of yellow) images of dauers incubated in the presence of BE. (F) Software analyzed image showing the area occupied by the dauers (red contour) and quantification of area and mean intensity of fluorescent beads inside the guts of dauers (green color contour) incubated in the presence of BE. Yellow contour shows either air bubbles or out-of focus parts of the worms, which were deleted manually and not considered in the image analysis. (G) Food signal activity in water and BE in arbitrary units. (H) Example of normalized food signal activity of the experiment that included images from Fig 1.
(TIF)

S2 Fig Food signal activity of the HPLC fractions. BE dissolved in 5 mM NaOH was considered as 100% as it had the highest activity. Food signal activities of all the conditions were normalized to BE in 5 mM NaOH.
(TIF)

S3 Fig HPLC separation of NAD^+^ dissolved in water. (A) HPLC-MS chromatogram of 3 mM NAD^+^ separated on C18 column (ES-). (B) Representative composite images showing the bright field signal (in shades of gray) and fluorescence signal from ingested beads (in shades of yellow) of dauers incubated with HPLC fractions dissolved either in water or 5 mM NaOH solution. Note the activity displayed by the fraction 25–30 min dissolved in 5 mM NaOH.
(TIF)

S4 Fig HPLC separation of NAD^+^ incubated in alkaline solution. (A) HPLC-MS chromatogram of 3 mM NAD^+^ incubated for 5.5 hours (time of the bioassay) in 5 mM NaOH and then separated on C18 column (ES-). (B) Representative composite images showing the bright field signal (in shades of gray) and fluorescence signal from ingested beads (in shades of yellow) of dauers incubated with HPLC fractions dissolved either in water or 5 mM NaOH solution. Note the activity displayed by the fraction 25–30 min dissolved in 5 mM NaOH.
(TIF)

S5 Fig Summary of mouth opening observed by electron microscopy. (TIF)

S6 Fig Serotonin does not cause the opening of the mouth in *tph-1* dauers. Representative images of the mouth in *tph-1(n4622)* dauers exposed to serotonin in (A) water and (B) borate buffer. Scale bars show 5 μm in all micrographs.
(TIF)
