# Supplementary figures and images for: NAD+ Is a Food Component That Promotes Exit from Dauer Diapause in Caenorhabditis elegans
Source: PLoS One. 2016 Dec 1;11(12):e0167208. doi: 10.1371/journal.pone.0167208 (PMC5132307; doi:10.1371/journal.pone.0167208)

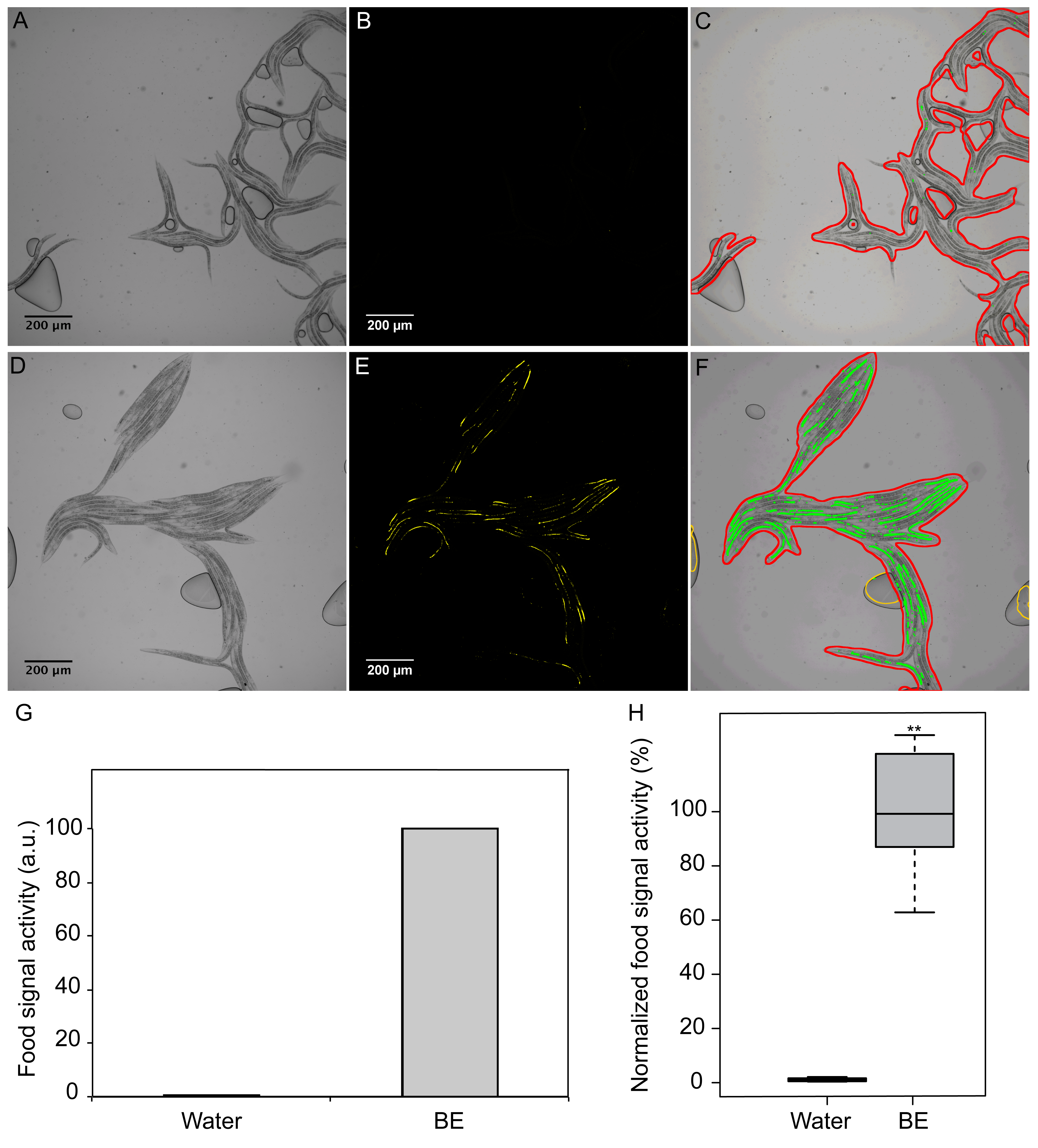

Supplement: S1 Fig — Images the same as in Fig 1 were used to exemplify food signal activity quantification procedure. (A) Bright field and (B) fluorescent (in shades of yellow) images of dauers incubated in water. (C) Software analyzed image showing the area occupied by the dauers (red contour) and quantification of area and mean intensity of fluorescent beads inside the guts of dauers (green color contour) incubated in water. (D) Bright field and (E) fluorescent (in shades of yellow) images of dauers incubated in the presence of BE. (F) Software analyzed image showing the area occupied by the dauers (red contour) and quantification of area and mean intensity of fluorescent beads inside the guts of dauers (green color contour) incubated in the presence of BE. Yellow contour shows either air bubbles or out-of focus parts of the worms, which were deleted manually and not considered in the image analysis. (G) Food signal activity in water and BE in arbitrary units. (H) Example of normalized food signal activity of the experiment that included images from Fig 1. (TIF) [file pone.0167208.s001.tif]

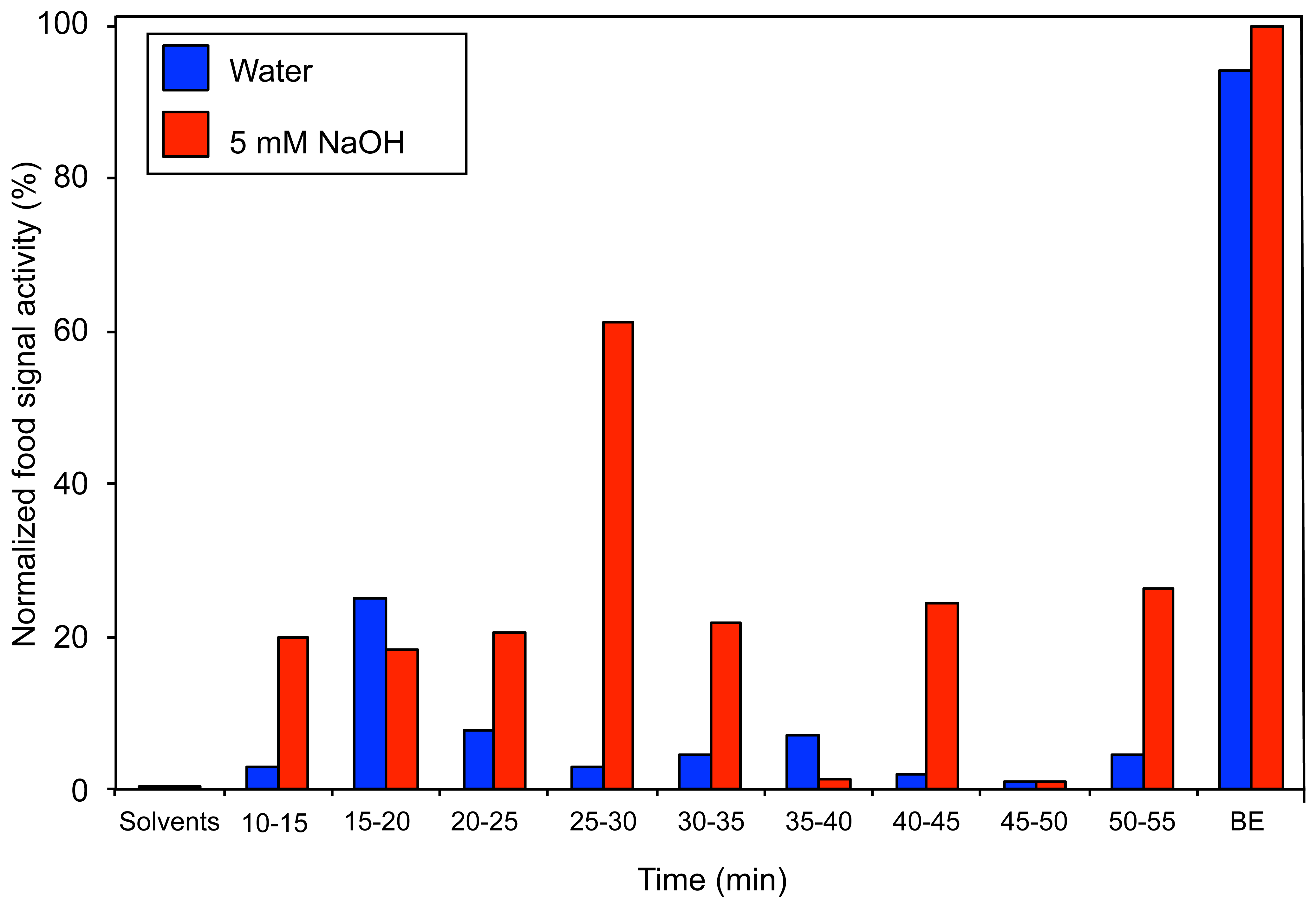

Supplement: S2 Fig — BE dissolved in 5 mM NaOH was considered as 100% as it had the highest activity. Food signal activities of all the conditions were normalized to BE in 5 mM NaOH. (TIF) [file pone.0167208.s002.tif]

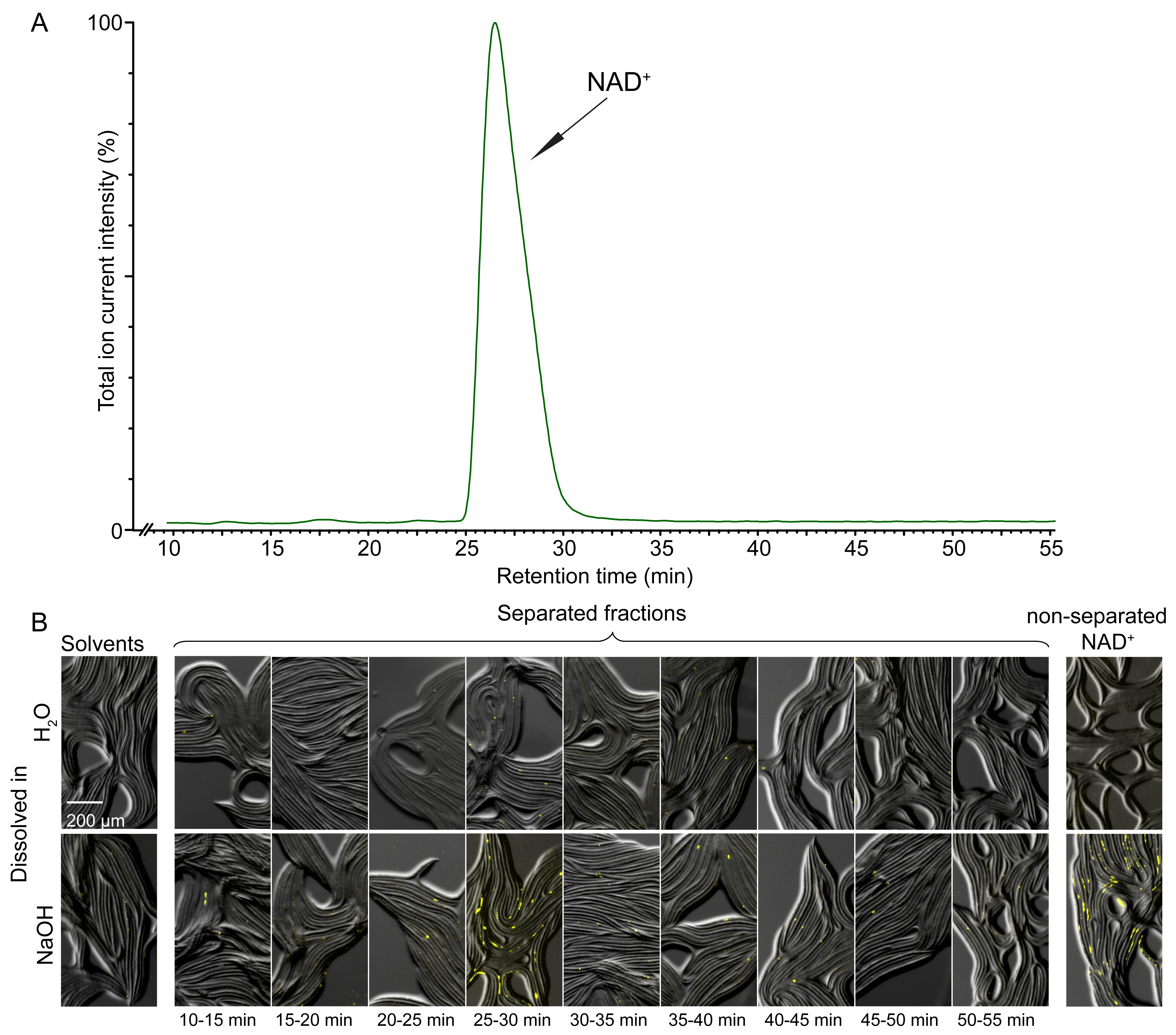

Supplement: S3 Fig — (A) HPLC-MS chromatogram of 3 mM NAD+ separated on C18 column (ES-). (B) Representative composite images showing the bright field signal (in shades of gray) and fluorescence signal from ingested beads (in shades of yellow) of dauers incubated with HPLC fractions dissolved either in water or 5 mM NaOH solution. Note the activity displayed by the fraction 25–30 min dissolved in 5 mM NaOH. (TIF) [file pone.0167208.s003.tif]

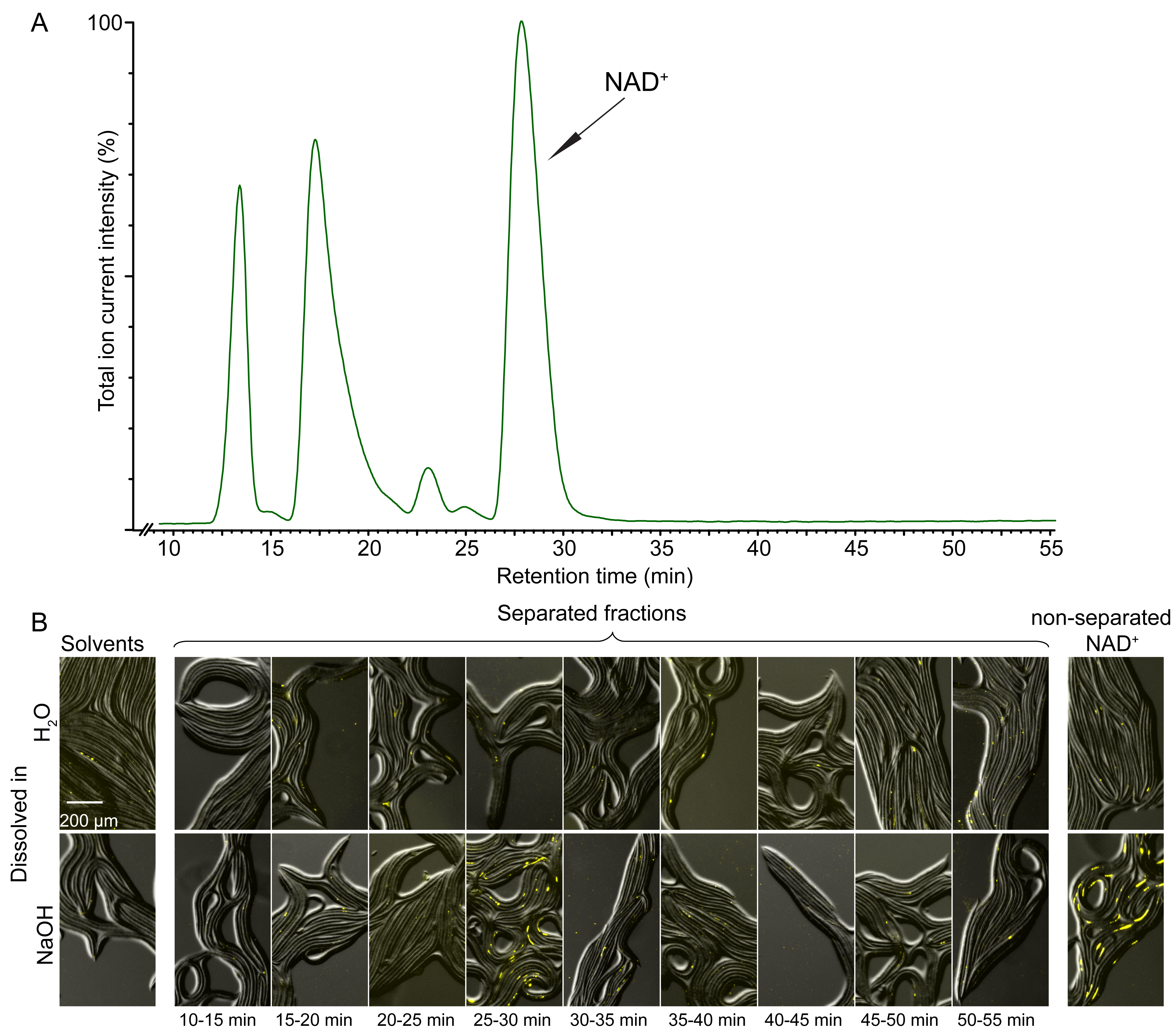

Supplement: S4 Fig — (A) HPLC-MS chromatogram of 3 mM NAD+ incubated for 5.5 hours (time of the bioassay) in 5 mM NaOH and then separated on C18 column (ES-). (B) Representative composite images showing the bright field signal (in shades of gray) and fluorescence signal from ingested beads (in shades of yellow) of dauers incubated with HPLC fractions dissolved either in water or 5 mM NaOH solution. Note the activity displayed by the fraction 25–30 min dissolved in 5 mM NaOH. (TIF) [file pone.0167208.s004.tif]

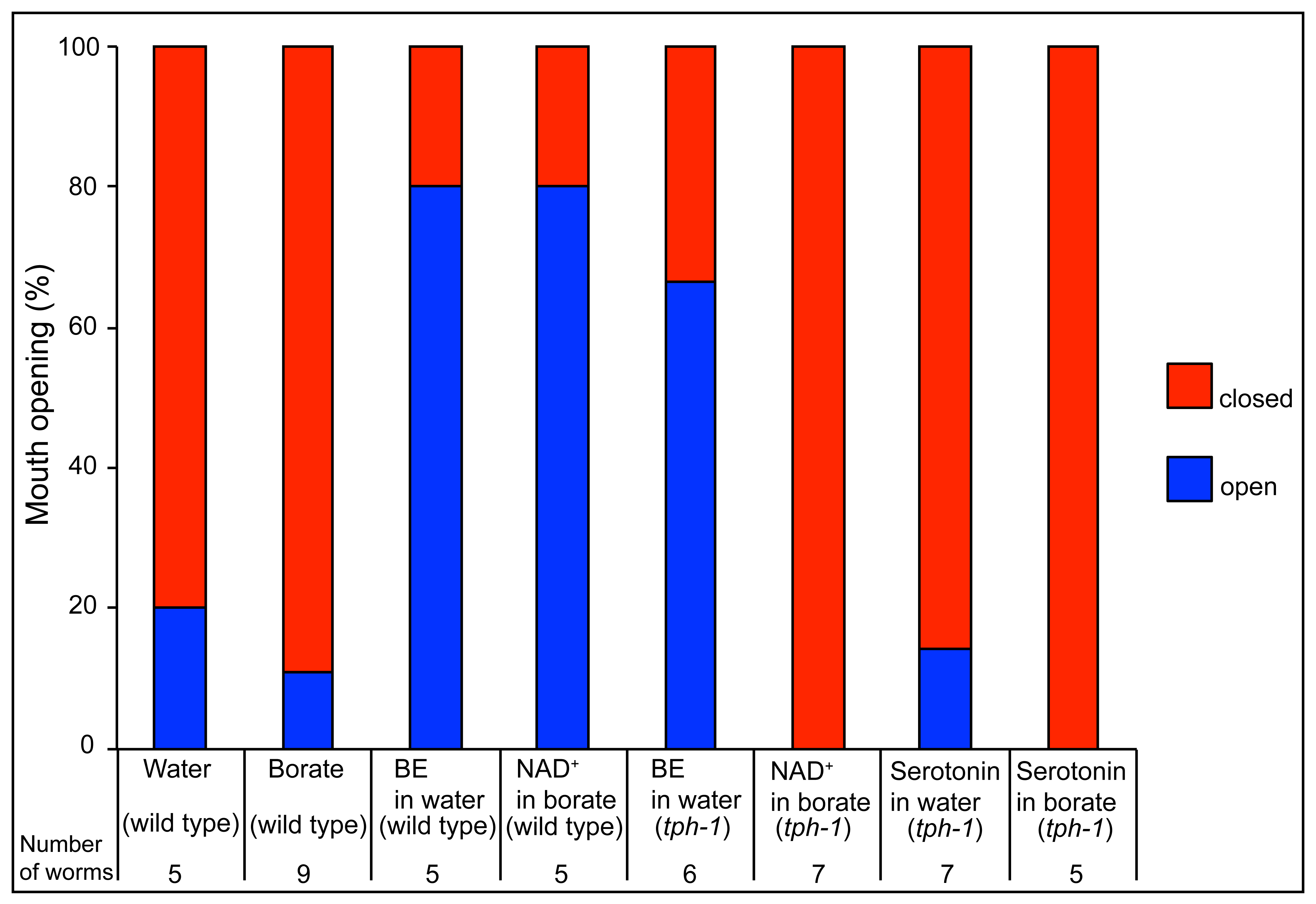

Supplement: S5 Fig — (TIF) [file pone.0167208.s005.tif]

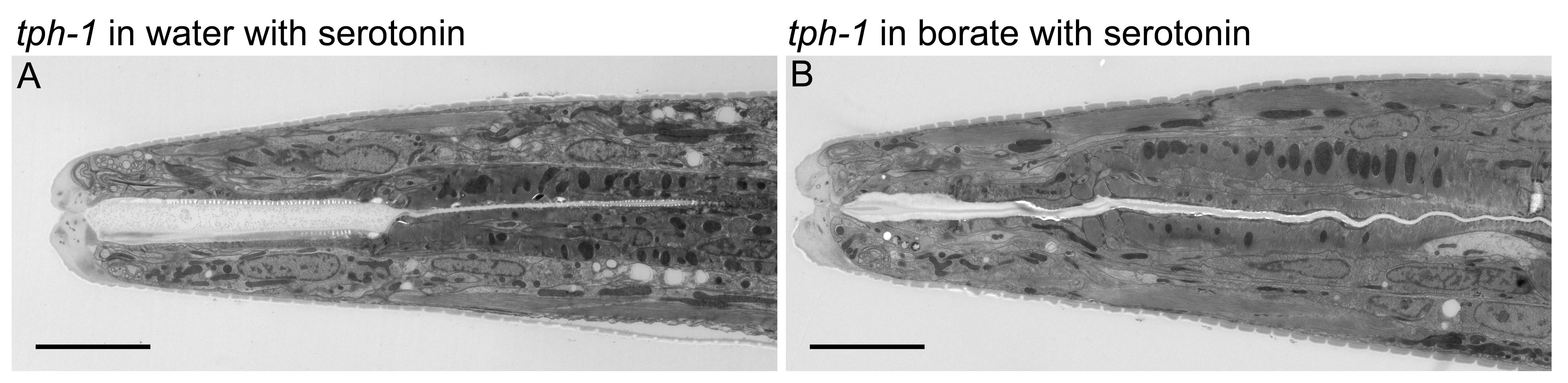

Supplement: S6 Fig — Representative images of the mouth in tph-1(n4622) dauers exposed to serotonin in (A) water and (B) borate buffer. Scale bars show 5 μm in all micrographs. (TIF) [file pone.0167208.s006.tif]
